# Supplementary material for: Multimodal mismatch responses in mouse auditory cortex
Source: eLife. 2025 Feb 10;13:RP95398. doi: 10.7554/eLife.95398 (PMC11810104; doi:10.7554/eLife.95398)
Supplement: Supplementary file 1. — We used hierarchical bootstrap (Saravanan et al., 2020) or a correlation coefficient for all comparisons. [file elife-95398-supp1.docx]

| **Figure** | **Comparison** | **Test** | **N_1_** | **N_2_** | **Unit** | **P-value** |
| --- | --- | --- | --- | --- | --- | --- |
| **Figure 1H** | Sound evoked response vs 0 | Hierarchical bootstrap | 7637 (60 fields of view, 17 mice) | - | Neurons | gray: n.s, black: p<0.05). |
| **Figure 1K** | Grating evoked response vs 0 | Hierarchical bootstrap | 7637 (60 fields of view, 17 mice) | - | Neurons | gray: n.s, black: p<0.05). |
| **Figure 1N** | Running onset response vs 0 | Hierarchical bootstrap | 7637 (60 fields of view, 17 mice) | - | Neurons | gray: n.s, black: p<0.05). |
| **Figure 2D** | AM mismatch vs sound playback halt response | Hierarchical bootstrap | 4755 (38 fields of view, 12 mice) | 4755 (38 fields of view, 12 mice) | Neurons | gray: n.s, black: p<0.05). |
| **Figure 2E** | Sound response vs running onset response of AM mismatch responsive neurons | - | 238 (33 fields of view, 12 mice) | 238 (33 fields of view, 12 mice) | Neurons | - |
| **Figure 2F** | Sound response of AM mismatch responsive neurons vs that of the remainder of the population | Hierarchical bootstrap | 238 (33 fields of view, 12 mice) | 4517 (38 fields of view, 12 mice) | Neurons | 0.0091 |
|  | Running onset response of AM mismatch responsive neurons vs that of the remainder of the population | Hierarchical bootstrap | 238 (33 fields of view, 12 mice) | 4517 (38 fields of view, 12 mice) | Neurons | 0.32 |
| **Figure 2H** | Running onset response in closed vs open loop session | Hierarchical bootstrap | 2407 neurons (22 fields of view, 10 mice) | 2407 neurons (22 fields of view, 10 mice) | Neurons | gray: n.s, black: p<0.05). |
| **Figure 3D** | VM mismatch vs playback halt response | Hierarchical bootstrap | 5688 (47 fields of view, 15 mice) | 5688 (47 fields of view, 15 mice) | Neurons | gray: n.s, black: p<0.05). |
| **Figure 3E** | Grating response vs running onset response of VM mismatch responsive neurons | - | 284 (43 fields of view, 15 mice) | 284 (43 fields of view, 15 mice) | Neurons | - |
| **Figure 3F** | Grating response of VM mismatch responsive neurons vs that of the remainder of the population | Hierarchical bootstrap | 284 (43 fields of view, 15 mice) | 5404 (47 fields of view, 15 mice) | Neurons | 0.23 |
|  | Running onset response of VM mismatch responsive neurons vs that of the remainder of the population | Hierarchical bootstrap | 284 (43 fields of view, 15 mice) | 5404 (47 fields of view, 15 mice) | Neurons | 0.45 |
| **Figure 3H** | Running onset response in closed vs open loop session | Hierarchical bootstrap | 2316 (17 fields of view, 9 mice) | 2316 (17 fields of view, 9 mice) | Neurons | gray: n.s, black: p<0.05). |
| **Figure 4C** | Response to concurrent [AM + VM] mismatches vs 0 | Hierarchical bootstrap | 3289 (26 fields of view, 10 mice) | 3289 (26 fields of view, 10 mice) | Neurons | gray: n.s, black: p<0.05). |
| **Figure 4D** | Response to concurrent [AM + VM] mismatches vs response to AM mismatches | Hierarchical bootstrap | 3289 (26 fields of view, 10 mice) | 3289 (26 fields of view, 10 mice) | Neurons | gray: n.s, black: p<0.05). |
|  | Response to concurrent [AM + VM] mismatches vs response to VM mismatches | Hierarchical bootstrap | 3289 (26 fields of view, 10 mice) | 3289 (26 fields of view, 10 mice) | Neurons | gray: n.s, black: p<0.05). |
| **Figure 4E** | Response to concurrent [AM + VM] mismatches vs the linear sum of the responses evoked by [AM] and [VM] mismatches presented in isolation | Hierarchical bootstrap | 3289 (26 fields of view, 10 mice) | 3289 (26 fields of view, 10 mice) | Neurons | gray: n.s, black: p<0.05). |
| **Figure 4F** | Correlation of the responses to the concurrent [AM+VM] mismatches against the linear sum of the responses evoked by [AM] and [VM] mismatches presented in isolation | Correlation | 3289 (26 fields of view, 10 mice) | 3289 (26 fields of view, 10 mice) | Neurons | p < 0.001  r = 0.31 |
| **Figure 1 – figure supplement 1A** | Sound evoked response during sitting vs running | Hierarchical Bootstrap | 4390 (33 fields of view, 14 mice) | 4390 (33 fields of view, 14 mice) | Neurons | gray: n.s, black: p<0.05). |
| **Figure 1 – figure supplement 1B** | Grating evoked response during sitting vs running | Hierarchical Bootstrap | 3701 (28 fields of view, 10 mice) | 3701 (28 fields of view, 10 mice) | Neurons | gray: n.s, black: p<0.05). |
| **Figure 2 – figure supplement 1A** | Running speed upon AM mismatch vs sound playback halt | Hierarchical Bootstrap | 38 sessions (12 mice) | 38 sessions (12 mice) | Sessions | gray: n.s, black: p<0.05). |
|  | Running speed upon AM mismatch vs baseline | Hierarchical Bootstrap | 38 sessions (12 mice) | - | Sessions | 0.75 |
|  | Running speed upon sound playback halt vs baseline | Hierarchical Bootstrap | 38 sessions (12 mice) | - | Sessions | 0.49 |
| **Figure 2 – figure supplement 1B** | Pupil diameter upon AM mismatch vs sound playback halt | Hierarchical Bootstrap | 32 sessions (11 mice) | 32 sessions (11 mice) | Sessions | gray: n.s, black: p<0.05). |
|  | Pupil diameter upon AM mismatch vs baseline | Hierarchical Bootstrap | 32 sessions (11 mice) | - | Sessions | 0.52 |
|  | Pupil diameter upon sound playback halt vs baseline | Hierarchical Bootstrap | 32 sessions (11 mice) | - | Sessions | 0.53 |
| **Figure 2 – figure supplement 1C** | AM mismatch on the 1^st^ recording day vs 0 | Hierarchical Bootstrap | 1271 (12 fields of view, 12 mice) | - | Neurons | gray: n.s, black: p<0.05). |
|  | AM mismatch on the 2^nd^ recording day vs 0 | Hierarchical Bootstrap | 904 (10 fields of view, 10 mice) | - | Neurons | gray: n.s, black: p<0.05). |
| **Figure 2 – figure supplement 1D** | Running speed in AM closed loop sessions vs that in open loop | Hierarchical Bootstrap | 38 sessions (12 mice) | 38 sessions (12 mice) | Sessions | 0.47 |
| **Figure 2 – figure supplement 1E** | Sound playback halt response during sitting vs running | - | 4017 (33 fields of view, 12 mice) | 1878 (17 fields of view, 9 mice) | Neurons | - |
| **Figure 2 – figure supplement 1F** | Correlation of the responses to AM mismatch and sound playback halt | Correlation | 4755 (38 fields of view, 12 mice) | 4755 (38 fields of view, 12 mice) | Neurons | p < 0.001  r = 0.66 |
| **Figure 2 – figure supplement 2** | Response to sound of the top 10% AM mismatch responsive neurons vs that of the remainder of the population | Hierarchical bootstrap | 476 (36 fields of view, 12 mice) | 4279 (38 fields of view, 12 mice) | Neurons | 0.0004 |
|  | Response to running onsets of the top 10% AM mismatch responsive neurons vs that of the remainder of the population | Hierarchical bootstrap | 476 (36 fields of view, 12 mice) | 4279 (38 fields of view, 12 mice) | Neurons | 0.48 |
|  | Response to sound of the top 20% AM mismatch responsive neurons vs that of the remainder of the population | Hierarchical bootstrap | 951 (38 fields of view, 12 mice) | 3804 (38 fields of view, 12 mice) | Neurons | 0.0002 |
|  | Response to running onsets of the top 20% AM mismatch responsive neurons vs that of the remainder of the population | Hierarchical bootstrap | 951 (38 fields of view, 12 mice) | 3804 (38 fields of view, 12 mice) | Neurons | 0.38 |
| **Figure 3 – figure supplement 1A** | Running speed upon VM mismatch vs playback halt | Hierarchical bootstrap | 47 sessions (15 mice) | 47 sessions (15 mice) | Sessions | gray: n.s, black: p<0.05). |
|  | Running speed upon VM mismatch vs baseline | Hierarchical bootstrap | 47 sessions (15 mice) | - | Sessions | 0.45 |
|  | Running speed upon playback halt vs baseline | Hierarchical bootstrap | 47 sessions (15 mice) | - | Sessions | 0.38 |
| **Figure 3 – figure supplement 1B** | Pupil diameter upon VM mismatch vs playback halt | Hierarchical bootstrap | 32 sessions (11 mice) | 32 sessions (11 mice) | Sessions | gray: n.s, black: p<0.05). |
|  | Pupil diameter upon VM mismatch vs baseline | Hierarchical bootstrap | 32 sessions (11 mice) | - | Sessions | 0.51 |
|  | Pupil diameter upon playback halt vs baseline | Hierarchical bootstrap | 32 sessions (11 mice) | - | Sessions | 0.52 |
| **Figure 3 – figure supplement 1C** | Correlation of the responses to VM mismatch and visual flow playback halt | Correlation | 5688 (47 fields of view, 15 mice) | 5688 (47 fields of view, 15 mice) | Neurons | p = 0.41  r = 0.01 |
| **Figure 4 – figure supplement 1A** | Running speed upon concurrent [AM + VM] mismatch vs baseline | Hierarchical bootstrap | 26 sessions (10 mice) | - | Sessions | 0.37 |
| **Figure 4 – figure supplement 1B** | Pupil diameter upon concurrent [AM + VM] mismatch vs baseline | - | 26 sessions (10 mice) | - | Sessions | 0.39 |
| **Figure 4 – figure supplement 1C** | Response to concurrent [AM + VM] mismatches vs the linear sum of the responses evoked by [AM] and [VM] mismatches presented in isolation | Hierarchical bootstrap | 3289 (26 fields of view, 10 mice) | 3289 (26 fields of view, 10 mice) | Neurons | gray: n.s, black: p<0.05). |
